# COSMIC (Cohort Studies of Memory in an International Consortium): An international consortium to identify risk and protective factors and biomarkers of cognitive ageing and dementia in diverse ethnic and sociocultural groups

**DOI:** 10.1186/1471-2377-13-165

**Published:** 2013-11-06

**Authors:** Perminder S Sachdev, Darren M Lipnicki, Nicole A Kochan, John D Crawford, Kenneth Rockwood, Shifu Xiao, Juan Li, Xia Li, Carol Brayne, Fiona E Matthews, Blossom CM Stephan, Richard B Lipton, Mindy J Katz, Karen Ritchie, Isabelle Carrière, Marie-Laure Ancelin, Sudha Seshadri, Rhoda Au, Alexa S Beiser, Linda CW Lam, Candy HY Wong, Ada WT Fung, Ki Woong Kim, Ji Won Han, Tae Hui Kim, Ronald C Petersen, Rosebud O Roberts, Michelle M Mielke, Mary Ganguli, Hiroko H Dodge, Tiffany Hughes, Kaarin J Anstey, Nicolas Cherbuin, Peter Butterworth, Tze Pin Ng, Qi Gao, Simone Reppermund, Henry Brodaty, Kenichi Meguro, Nicole Schupf, Jennifer Manly, Yaakov Stern, Antonio Lobo, Raúl Lopez-Anton, Javier Santabárbara

**Affiliations:** 1 Centre for Healthy Brain Ageing, University of New South Wales, Sydney, Australia; 2 Dementia Collaborative Research Centre, University of New South Wales, Sydney, Australia; 3 Department of Medicine (Geriatric Medicine & Neurology), Dalhousie University, Halifax, NS, Canada; 4 Department of Geriatric Psychiatry, Shanghai Mental Health Center, Shanghai Jiaotong University School of Medicine, Shanghai, China; 5 Institute of Psychology, Chinese Academy of Sciences, Beijing, China; 6 Department of Public Health and Primary Care, Cambridge University, Cambridge, UK; 7 MRC Biostatistics Unit, Institute of Public Health, Cambridge, UK; 8 Institute of Health and Society, Newcastle University, Newcastle upon Tyne, UK; 9 Saul B. Korey Department of Neurology; Albert Einstein College of Medicine, Yeshiva University, New York, NY, USA; 10 Department of Epidemiology and Population Health, Albert Einstein College of Medicine, Yeshiva University, New York, NY, USA; 11 Inserm, U1061 Nervous System Pathologies: Epidemiological and Clinical Research, La Colombière Hospital, Montpellier Cedex 5, France; 12 Université de Montpellier 1, Montpellier, France; 13 Faculty of Medicine, Imperial College, St Mary’s Hospital, London, UK; 14 Department of Neurology, Boston University School of Medicine, Boston, MA, USA; 15 Department of Biostatistics, Boston University School of Public Health, Boston, MA, USA; 16 Department of Psychiatry, The Chinese University of Hong Kong, Hong Kong, SAR, China; 17 Department of Psychiatry, Tai Po Hospital, Hong Kong, SAR, China; 18 Department of Neuropsychiatry, Seoul National University Bundang Hospital, Seongnam, Korea; 19 Department of Psychiatry, Seoul National University College of Medicine, Seoul, Korea; 20 Department of Brain and Cognitive Sciences, Seoul National University College of Natural Sciences, Seoul, Korea; 21 Mayo Clinic College of Medicine, Rochester, MN, USA; 22 Department of Psychiatry, University of Pittsburgh School of Medicine, Pittsburgh, PA, USA; 23 Department of Neurology, University of Pittsburgh School of Medicine, Pittsburgh, PA, USA; 24 Department of Epidemiology, University of Pittsburgh Graduate School of Public Health, Pittsburgh, PA, USA; 25 Department of Neurology, Oregon Health and Science University, Portland, OR, USA; 26 Department of Neurology, University of Michigan, Ann Arbor, MI, USA; 27 Centre for Research on Ageing, Health and Wellbeing, College of Medicine, Biology and Environment, The Australian National University, Canberra, Australia; 28 Gerontology Research Programme, Department of Psychological Medicine, Yong Loo Lin School of Medicine, National University of Singapore, Singapore; 29 Department of Geriatric Behavioral Neurology, Tohoku University Graduate School of Medicine, Sendai, Japan; 30 The Taub Institute for Research in Alzheimer’s Disease and the Aging Brain, Columbia University, New York, NY, USA; 31 The Gertrude H. Sergievsky Center, Columbia University, New York, NY, USA; 32 The Division of Epidemiology, Joseph P. Mailman School of Public Health, Columbia University, New York, NY, USA; 33 The Department of Neurology, Columbia University, New York, NY, USA; 34 Department of Medicine and Psychiatry, Universidad de Zaragoza, Zaragoza, Spain; 35 Centro de Investigación Biomédica en Red de Salud Mental (CIBERSAM), Ministry of Science and Innovation, Madrid, Spain; 36 Instituto de Investigación Sanitaria de Aragón (IIS Aragón), Zaragoza, Spain; 37 Department of Psychology and Sociology, Universidad de Zaragoza, Zaragoza, Spain; 38 Department of Preventive Medicine and Public Health, Universidad de Zaragoza, Zaragoza, Spain

**Keywords:** Cohort studies, Cognitive ageing, Data harmonisation, Dementia, International consortium, Mild cognitive impairment

## Abstract

**Background:**

A large number of longitudinal studies of population-based ageing cohorts are in progress internationally, but the insights from these studies into the risk and protective factors for cognitive ageing and conditions like mild cognitive impairment and dementia have been inconsistent. Some of the problems confounding this research can be reduced by harmonising and pooling data across studies. COSMIC (Cohort Studies of Memory in an International Consortium) aims to harmonise data from international cohort studies of cognitive ageing, in order to better understand the determinants of cognitive ageing and neurocognitive disorders.

**Methods/Design:**

Longitudinal studies of cognitive ageing and dementia with at least 500 individuals aged 60 years or over are eligible and invited to be members of COSMIC. There are currently 17 member studies, from regions that include Asia, Australia, Europe, and North America. A Research Steering Committee has been established, two meetings of study leaders held, and a website developed. The initial attempts at harmonising key variables like neuropsychological test scores are in progress.

**Discussion:**

The challenges of international consortia like COSMIC include efficient communication among members, extended use of resources, and data harmonisation. Successful harmonisation will facilitate projects investigating rates of cognitive decline, risk and protective factors for mild cognitive impairment, and biomarkers of mild cognitive impairment and dementia. Extended implications of COSMIC could include standardised ways of collecting and reporting data, and a rich cognitive ageing database being made available to other researchers. COSMIC could potentially transform our understanding of the epidemiology of cognitive ageing, and have a world-wide impact on promoting successful ageing.

## Background

The ageing of our populations, with the increasing prevalence of physical and cognitive disorders associated with age, poses a major burden on society [1]. Making an impact on this disability burden requires an understanding of the risk and protective factors for age-related cognitive decline, frailty and chronic disease. The optimal approach to study this involves the longitudinal examination of population-based ageing cohorts. There are many such studies currently ongoing internationally, but there is considerable inconsistency in the results produced [2] and further systematic examination of the existing evidence is required to determine findings which are robust.

Some of the variation in prevalence rates and risk factors identified across studies may be associated with regional and/or ethnic differences. For example, rates of non-amnestic mild cognitive impairment (MCI) are reported to be higher in blacks than whites from a similar geographical location, even when controlling for sex and education [3]. However, a significant proportion of the variance between studies is likely attributable to differences in methodology, including differences in the assessment tools and performance criteria used for diagnosing cognitive disorders. Indeed, small but theoretically valid changes to how objective cognitive impairment was operationally defined led to greatly elevated prevalence rates, from 4% to 70% [4]. Similarly, using different criteria for the diagnosis of dementia can result in vastly different prevalence figures [5].

There are a number of approaches for overcoming methodological differences and other sources of heterogeneity so that studies can be more accurately compared and true differences identified. These include the use of standardised protocols, meta-analysis, and harmonisation of data [6]. The use of standardised or even similar protocols is a rare feature of existing collaborations (the 10/66 Dementia Research Group is one exception [7]), and meta-analysis is limited to published results. In contrast, data harmonisation offers the potential to explore both existing and novel research questions by a cost-effective use of previously-collected data.

Harmonising data across studies to create a single, large database helps to minimise the influence of both study-level (e.g., methodology) and individual level (e.g., demographic) factors, while also enabling these to be explored as potential contributors to differences in results [8,9]. Other advantages include increased statistical power for detecting effects, and the inherent replication and enhanced generalisability associated with using heterogeneous samples and methodologies [8].

COSMIC (Cohort Studies of Memory in an International Consortium) is a recently established endeavour that aims to bring together cohort studies of cognitive ageing internationally in order to facilitate a better understanding of the determinants of cognitive ageing and neurocognitive disorders. The two main objectives of this project are to:

1. Harmonise shared, non-identifiable data from cohort studies that longitudinally examine change in cognitive function and the development of dementia in older individuals (60+ years).

2. Perform joint or mega-analyses using combined, harmonised data sets that yield collated results with enhanced statistical power, in addition to comparisons across geographical regions, ethnicities and sociocultural groups.

Other collaborations bringing together cohort studies of ageing include the genetics-focused CHARGE (Cohorts for Heart and Aging Research in Genomic Epidemiology) [10] and ENIGMA (Enhancing NeuroImaging Genetics through Meta-Analysis) [11]. Consortia with a particular interest in cognitive ageing include the UK-based HALCyon (Healthy Ageing across the Life Course) [12], the primarily Europe-based CHANCES (Consortium on Health and Ageing: Network of Cohorts in Europe and the United States) [13], the Australian-based DYNOPTA (Dynamic Analyses to Optimise Ageing) [14], and the IALSA (Integrative Analysis of Longitudinal Studies on Aging) network [15], which has member studies from Europe, North America and Australia. None of these consortia have any studies from Asia, where the current and future number of people with dementia is estimated to be greater than that of Europe and the Americas combined [1]. COSMIC hopes to distinguish itself by being a truly international effort comprising studies with a clinical and biomedical focus from Asia, Europe, the Americas, and Oceania. COSMIC was established in 2012, with progress reported in 2013 [16].

## Methods/design

### Membership

Studies are eligible to participate in COSMIC if they meet the following membership criteria:

1. Are epidemiological, and therefore population-based.

2. Have a minimum sample size of 500.

3. Examine individuals aged 60 years and over.

4. Are longitudinal, with a minimum of two assessments.

5. Include assessment of cognitive function as an important, if not central, objective.

6. The outcome measures include dementia and/or cognitive impairment and/or cognitive decline.

Official enrolment in COSMIC involves a lead investigator having signed a memorandum of understanding that entails a willingness to share non-identifiable raw and/or processed data for joint or mega-analyses. Studies that, for institutional or other reasons, are unable to provide raw and/or processed data may participate in COSMIC as provisional members, if willing to provide results of in-house analyses conducted using COSMIC protocols. At the time of writing there are 14 officially enrolled and 3 provisional members of COSMIC. These studies, and their key demographic characteristics, are shown in Table 1. It is intended that the overall sample size and range of geographical regions and ethnicities represented be extended even further, and thus we ask that any study meeting the eligibility criteria consider contacting us to become a member of COSMIC. Studies from Africa, South America and Eastern Europe are particularly encouraged to join.

**Table 1:** COSMIC member studies

**Study**	**Country**	**Sample size**	**Age range**	**Males (%)**	**Main races/ethnicities**	**Start and end date**	**Key reference(s)**
Canadian Study of Health and Aging (CSHA)	Canada	10263	65-102	43	Caucasian	1991-2002	CSHA Working Group (1994) [17]
Chinese Longitudinal Ageing Study (CLAS)^*^	China	3514	60+	44	Chinese	2010-	Xiao et al. (2013) [18]
Cognitive Function and Ageing Studies (CFAS)^†^	UK	13004	65+	40	Caucasian	1991-	Brayne et al. (2006) [19]
Einstein Aging Study (EAS)^*^	USA	1956	66-104	39	Caucasian/African American	1993-	Katz et al. (2012) [3]
Etude Santé Psychologique Prévalence Risques et Traitement (ESPRIT)^*^	France	2268	65+	42	Not recorded	1999-	Ritchie et al. (2010) [20]
Framingham Heart Study (FHS)^†^	USA	15328^‡^	5+	50	Caucasian	1948-	Dawber & Kannel (1958) [21]; Feinleib et al. (1975) [22]; Splansky et al. (2007) [23]
Hong Kong Memory and Ageing Prospective Study (HK-MAPS)^*^	Hong Kong	787	60+	46	Chinese	2005-	Wong et al. (2013) [24]
Korean Longitudinal Study on Cognitive Aging and Dementia (KLOSCAD)	South Korea	6479	60+	44	Korean	2009-	Kim et al. (2013) [25]
Mayo Clinic Study of Aging (MCSA)^†^	USA	4000	50-89	50	Caucasian	2004-	Roberts et al. (2008) [26]
Monongahela Valley Independent Elders Survey (MoVIES)^*^	USA	1681	65+	42	Caucasian	1987-2002	Ganguli et al. (2000) [27]
Personality and Total Health (PATH) Through Life Project^*^	Australia	2551	60-64	52	Caucasian	2001-	Anstey et al. (2012) [28]
Singapore Longitudinal Ageing Studies (SLAS) I and II^*^	Singapore	5748	54-98	37	Chinese	2003-	Feng et al. (2010, 2013) [29,30]
Sydney Memory and Ageing Study (Sydney MAS)^*^	Australia	1037	70+	45	Caucasian	2005-	Sachdev et al. (2010) [31]
Tajiri Project	Japan	1654	65+	42	Japanese	1998-2005	Meguro et al. (2002) [32]
Washington Heights Inwood and Columbia Aging Project (WHICAP)^*^	USA	4577	63-103	32	Hispanic/African American/Caucasian	1989-	Tang et al. (2001) [33]
ZARADEMP Project (ZARAgoza DEMentia DEPression Project)^*^	Spain	4803	55+	42	Caucasian	1994-	Lobo et al. (2005) [34]

^*^Data for the first project have been made available.

^†^Provisional member.

^‡^Including 3 generations (Original cohort, Offspring, Grandchildren) and separate Omni cohort of 900 ethnic minority participants.

### Organisation

COSMIC has a Research Steering Committee comprising one representative from each participating study, generally the lead investigator or a delegate. The primary functions of the Research Steering Committee are:

1. To develop guidelines for the inclusion and exclusion of studies.

2. To provide rules of participation and guidelines for the roles and responsibilities of the participating studies.

3. To approve Workgroups.

4. To select topics of interest.

5. To provide overall analytic strategies.

6. To develop rules for publication, including authorship.

7. To develop rules for the protection of intellectual property, when relevant.

8. To seek funds to support COSMIC.

### Meetings

An initial meeting of many (now member) study leaders on July 16, 2012 in Vancouver supported the official establishment of COSMIC. Potential projects, both initial and more long-term, and the steps needed to progress these were among the topics discussed. A subsequent meeting comprising many of the Research Steering Committee members was held on July 15, 2013 in Boston.

### Website

A website has been established that contains a description of COSMIC and summaries of the member studies (http://www.cheba.unsw.edu.au/group/cosmic). This website is intended to serve as an avenue for presenting and preserving COSMIC project protocols and results, and will potentially house data restricted by password to COSMIC members. The Sydney team are currently responsible for the development and maintenance of the COSMIC website.

### Ethics

The overall COSMIC project has been approved by the Human Research Ethics Committee of the University of New South Wales, Sydney. Member studies are responsible for obtaining approval (if considered necessary) from their local institutional review board for the sharing of data. However, de-identified data are not considered Protected Health information by the National Institute of Health of the USA. A protocol for the de-identification of data has been developed.

## Discussion

### Challenges

General challenges facing large, international consortia have been previously described (e.g., by CHARGE [10]). These include a potential need for additional funding to prolong the use of study data beyond initial anticipations, and timely and effective communication among members across different countries.

More specific to COSMIC are challenges associated with harmonisation, many of which have also been previously described [9,35]. The major challenge of harmonisation stems from differences between studies in the measurement instruments used and/or differences in how questions from similar instruments are worded and responses provided and categorised, including the effect of language and culture. Attempts to maximise the number of studies contributing to a final dataset can require that complex information from some studies be simplified (e.g., converted from a continuous measure to a categorical scale). There is a potential reduction in validity involved in simplifying data, but there are mechanisms by which this can be tested and/or quantified [9].

Meeting the objectives of COSMIC will require various data types to be harmonised, but data relating to cognitive outcomes such as impairment and decline are likely to be the most challenging (i.e., more so than demographic and health-related variables). COSMIC member studies have operationally defined cognitive outcomes in vastly different ways. For example, for the purposes of diagnosing MCI, cognitive impairment has been variously defined as abnormal scores on the memory items of two cognitive status instruments (Mini-Mental State Examination and Geriatric Mental State Schedule) in the Zaragoza Dementia Depression Project [36], and as a score on any measure from a comprehensive neuropsychological battery 1.5 or more standard deviations below published normative values in the Sydney Memory and Ageing Study [31]. Different studies have used different neuropsychological test batteries, but even when similar cognitive tests have been used it is often the case that different versions have been used or the tests have been administered in a non-standard way. An added complication is the need to reconcile differences in the data while giving appropriate consideration to relevant demographic effects, including those associated with gender and education.

### First project

The aim of the first COSMIC project is to compare the baseline prevalence of MCI across the COSMIC member cohorts and the different regions and ethnicities represented by these. The project is currently underway, and is being coordinated by the Sydney team. A questionnaire was developed and promulgated, with the information provided guiding a subsequent request for data from the studies on:

1. Demographics.

2. Sample representativeness.

3. Neuropsychological test performance.

4. Functional test scores.

5. Memory/cognitive complain/concerns.

6. Criteria used for MCI.

The receipt of data was followed by communication with data managers and/or study leaders to clarify the nature of data (e.g., the particular neuropsychological test used or manner of administration) and/or to ask that further data be provided (e.g., the individual items from a functional test scale in addition to a total score originally provided). Data from 11 studies have been made available for this project (see Table 1), and there is a total sample size of more than 23,000 non-demented individuals aged 60 and older.

Some demographic variables have been harmonised. All studies provided age in years, and harmonising sex only required some recoding to a common scale (female = 0; male = 1). Education was less straightforward. A four-level categorical scale of the highest level of education achieved (Less than high school completion, High school completion, Technical or college diploma, University degree) was chosen as the most appropriate common measure, and to which various other categorical formats or years of formal education were transformed (see Additional file 1 for the protocol). Data were provided in the harmonised format by the studies themselves, or later transformed from the original variable by the project coordinators.

The next step will be to harmonise the data needed to make classifications of MCI. The participating studies have published widely varying rates of MCI, from as low as 3.2% for the Monongahela Valley Independent Elders Survey [37] to 34.8% for the Sydney Memory and Ageing Study [31]. Differences between the studies in how MCI diagnoses were made have undoubtedly contributed to the varying prevalence rates [4], and minimising this requires the harmonisation of data informing the four generally accepted criteria for MCI:

1. Absence of dementia.

2. No or minimal functional impairment.

3. Objective cognitive impairment.

4. Memory complaint or concern [38,39].

### Future projects

A number of future projects utilising COSMIC data are currently planned, and aim to make comparisons across COSMIC cohorts, countries and ethnic groups of:

1. Risk and protective factors for MCI.

2. Rates of cognitive decline.

3. Biomarkers (e.g., blood, genetic and MRI-derived) of MCI and dementia.

Many of the existing member studies have relevant data to contribute to these projects. It is expected that additional projects will address more refined and specific topics addressing the overall objectives of COSMIC. This could include identifying and comparing rates of decline within particular cognitive domains, and establishing associations between untreated hypertension or non-traditional risk factors and cognitive decline. Projects like these will be enabled and facilitated by growing the COSMIC membership base to ensure that there are sufficient relevant data on variables not collected by all studies.

### Extended Implications of COSMIC

The mechanisms for harmonising measures developed by COSMIC could produce standardised ways of collecting and reporting data that facilitate the comparability of longitudinal studies of ageing. This includes previous or existing studies, for which data could be reformatted and further analysed. It may also guide the choice of measures used or type of data collected by future studies, for which the capacity to directly compare results with those of many other cohorts would greatly enhance their interpretability and relevance.

There is also the potential for the COSMIC database to be made available to non-consortium researchers via the website, following consortia-based publications and with the approval of the Research Steering Committee. The scientific benefits of making large databases available to researchers worldwide are demonstrated by the more than 250 publications reported to have arisen from the sharing of ADNI (Alzheimer's Disease Neuroimaging Initiative) data across the internet [40].

With these, and potentially further extended implications and uses of COSMIC data, member studies can be confident that their data are being fully utilised and that they are contributing to a truly global effort to understand and combat the problems associated with cognitive ageing, MCI and dementia.

## Conclusion

The COSMIC project is a truly international effort to inform the epidemiology of cognitive disorders associated with advanced age by identifying risk factors and biomarkers that are common as well as unique. It has the potential to transform our understanding of the epidemiology of cognitive ageing and have a world-wide impact on promoting successful ageing.

## Abbreviations

COSMIC: Cohort Studies of Memory in an International Consortium; MCI: Mild cognitive impairment.

## Competing interests

The authors declare that they have no competing interests, except for Kenneth Rockwood: Founder and shareholder of DGI Clinical; Richard Lipton and Mindy Katz have received monies from Bristol Myers Squibb Inc.; Ronald Petersen is on the Data Monitoring Boards of Pfizer Inc. and Janssen Alzheimer Immunotherapy, and a consultant to GE Healthcare; Rosebud Roberts has received a grant from Abbott Laboratories; Yaakov Stern is on the Advisory Boards of Janssen LLC, Pfizer Inc., Alzheimer’s Association and AbbVie, and has received Research Support from Piramal Research.

## Authors’ contributions

PSS is the instigator and head of COSMIC, and together with DML drafted the manuscript. All authors contributed to their local study, contributed to critically revising the manuscript for important intellectual content, and approved the final version of the manuscript for submission.

## Pre-publication history

The pre-publication history for this paper can be accessed here:

http://www.biomedcentral.com/1471-2377/13/165/prepub

## Supplementary Material

Additional file 1 Protocol for harmonising education across COSMIC member studies participating in the first project. Click here for file

## References

[B1] Prince M Bryce R Albanese E Wimo A Ribeiro W Ferri CP The global prevalence of dementia: a systematic review and metaanalysis Alzheimers Dement 2013 9 63 75 e62 10.1016/j.jalz.2012.11.007 23305823

[B2] Plassman BL Williams JW Jr Burke JR Holsinger T Benjamin S Systematic review: factors associated with risk for and possible prevention of cognitive decline in later life Ann Intern Med 2010 153 182 193 10.7326/0003-4819-153-3-201008030-00258 20547887

[B3] Katz MJ Lipton RB Hall CB Zimmerman ME Sanders AE Verghese J Dickson DW Derby CA Age-specific and sex-specific prevalence and incidence of mild cognitive impairment, dementia, and Alzheimer dementia in blacks and whites: a report from the Einstein Aging Study Alzheimer Dis Assoc Disord 2012 26 335 343 10.1097/WAD.0b013e31823dbcfc 22156756 PMC3334445

[B4] Kochan NA Slavin MJ Brodaty H Crawford JD Trollor JN Draper B Sachdev PS Effect of different impairment criteria on prevalence of "objective" mild cognitive impairment in a community sample Am J Geriatr Psychiatry 2010 18 711 722 10.1097/JGP.0b013e3181d6b6a9 21491632

[B5] Erkinjuntti T Ostbye T Steenhuis R Hachinski V The effect of different diagnostic criteria on the prevalence of dementia N Engl J Med 1997 337 1667 1674 10.1056/NEJM199712043372306 9385127

[B6] Beer-Borst S Morabia A Hercberg S Vitek O Bernstein MS Galan P Galasso R Giampaoli S Houterman S McCrum E Obesity and other health determinants across Europe: the EURALIM project J Epidemiol Community Health 2000 54 424 430 10.1136/jech.54.6.424 10818117 PMC1731700

[B7] Prince M Ferri CP Acosta D Albanese E Arizaga R Dewey M Gavrilova SI Guerra M Huang Y Jacob KS The protocols for the 10/66 dementia research group population-based research programme BMC Public Health 2007 7 165 10.1186/1471-2458-7-165 17659078 PMC1965476

[B8] Hofer SM Piccinin AM Integrative data analysis through coordination of measurement and analysis protocol across independent longitudinal studies Psychol Methods 2009 14 150 164 19485626 10.1037/a0015566 PMC2773828

[B9] Schaap LA Peeters GM Dennison EM Zambon S Nikolaus T Sanchez-Martinez M Musacchio E van Schoor NM Deeg DJ European Project on OSteoArthritis (EPOSA): methodological challenges in harmonization of existing data from five European population-based cohorts on aging BMC Musculoskelet Disord 2011 12 272 10.1186/1471-2474-12-272 22122831 PMC3257208

[B10] CHARGE (Cohorts for Heart and Aging Research in Genomic Epidemiology) [http://web.chargeconsortium.com]

[B11] ENIGMA (Enhancing NeuroImaging Genetics through Meta-Analysis) [http://enigma.loni.ucla.edu]

[B12] HALCyon (Healthy Ageing across the Life Course) [http://www.halcyon.ac.uk]

[B13] CHANCES (Consortium on Health and Ageing: Network of Cohorts in Europe and the United States) [http://www.chancesfp7.eu]

[B14] Anstey KJ Byles JE Luszcz MA Mitchell P Steel D Booth H Browning C Butterworth P Cumming RG Healy J Cohort profile: The Dynamic Analyses to Optimize Ageing (DYNOPTA) project Int J Epidemiol 2010 39 44 51 10.1093/ije/dyn276 19151373 PMC2817088

[B15] IALSA (Integrative Analysis of Longitudinal Studies on Aging) [http://web.uvic.ca/~ilife]

[B16] Sachdev P Lipnicki D Kochan N Crawford J COSMIC: Cohort Studies of Memory in an International Consortium [abstract] Alzheimers Dement 2013 9 Suppl 4 P611

[B17] Canadian Study of Health and Aging Working Group Canadian study of health and aging: study methods and prevalence of dementia CMAJ 1994 150 899 913 8131123 PMC1486712

[B18] Xiao S Li J Tang M Chen W Bao F Wang H Wang Y Liu Y Wang Y Yuan Y Methodology of China’s national study on the evaluation, early recognition, and treatment of psychological problems in the elderly: the China Longitudinal Aging Study Shanghai Archives of Psychiatry 2013 25 91 97 24991140 10.3969/j.issn.1002-0829.2013.02.005 PMC4054537

[B19] Brayne C McCracken C Matthews FE Cohort profile: the Medical Research Council Cognitive Function and Ageing Study (CFAS) Int J Epidemiol 2006 35 1140 1145 10.1093/ije/dyl199 16980700

[B20] Ritchie K Carriere I Ritchie CW Berr C Artero S Ancelin ML Designing prevention programmes to reduce incidence of dementia: prospective cohort study of modifiable risk factors BMJ 2010 341 c3885 10.1136/bmj.c3885 20688841 PMC2917002

[B21] Dawber TR Kannel WB An epidemiologic study of heart disease: the Framingham study Nutr Rev 1958 16 1 4 13493903 10.1111/j.1753-4887.1958.tb00605.x

[B22] Feinleib M Kannel WB Garrison RJ McNamara PM Castelli WP The Framingham Offspring Study. Design and preliminary data Prev Med 1975 4 518 525 10.1016/0091-7435(75)90037-7 1208363

[B23] Splansky GL Corey D Yang Q Atwood LD Cupples LA Benjamin EJ D'Agostino RB Sr Fox CS Larson MG Murabito JM The Third Generation Cohort of the National Heart, Lung, and Blood Institute's Framingham Heart Study: design, recruitment, and initial examination Am J Epidemiol 2007 165 1328 1335 10.1093/aje/kwm021 17372189

[B24] Wong CH Leung GT Fung AW Chan WC Lam LC Cognitive predictors for five-year conversion to dementia in community-dwelling Chinese older adults Int Psychogeriatr 2013 25 1125 1134 10.1017/S1041610213000161 23544873

[B25] Kim TH Park JH Lee JJ Jhoo JH Kim BJ Kim JL Kim SG Youn JC Ryu SH Lee DY Overview of the Korean Longitudinal Study on Cognitive Aging and Dementia [abstract] Alzheimers Dement 2013 9 Suppl 4 626 627

[B26] Roberts RO Geda YE Knopman DS Cha RH Pankratz VS Boeve BF Ivnik RJ Tangalos EG Petersen RC Rocca WA The Mayo Clinic Study of Aging: design and sampling, participation, baseline measures and sample characteristics Neuroepidemiology 2008 30 58 69 10.1159/000115751 18259084 PMC2821441

[B27] Ganguli M Dodge HH Chen P Belle S DeKosky ST Ten-year incidence of dementia in a rural elderly US community population: the MoVIES Project Neurology 2000 54 1109 1116 10.1212/WNL.54.5.1109 10720283

[B28] Anstey KJ Christensen H Butterworth P Easteal S Mackinnon A Jacomb T Maxwell K Rodgers B Windsor T Cherbuin N Cohort profile: the PATH through life project Int J Epidemiol 2012 41 951 960 10.1093/ije/dyr025 21349904

[B29] Feng L Chong MS Lim WS Lee TS Collinson SL Yap P Ng TP Metabolic syndrome and amnestic mild cognitive impairment: singapore longitudinal ageing study-2 findings J Alzheimers Dis 2013 34 649 657 23246920 10.3233/JAD-121885

[B30] Feng L Gwee X Kua EH Ng TP Cognitive function and tea consumption in community dwelling older Chinese in Singapore J Nutr Health Aging 2010 14 433 438 10.1007/s12603-010-0095-9 20617284 PMC12880352

[B31] Sachdev PS Brodaty H Reppermund S Kochan NA Trollor JN Draper B Slavin MJ Crawford J Kang K Broe GA The Sydney Memory and Ageing Study (MAS): methodology and baseline medical and neuropsychiatric characteristics of an elderly epidemiological non-demented cohort of Australians aged 70–90 years Int Psychogeriatr 2010 22 1248 1264 10.1017/S1041610210001067 20637138

[B32] Meguro K Ishii H Yamaguchi S Ishizaki J Shimada M Sato M Hashimoto R Shimada Y Meguro M Yamadori A Prevalence of dementia and dementing diseases in Japan: the Tajiri project Arch Neurol 2002 59 1109 1114 10.1001/archneur.59.7.1109 12117358

[B33] Tang MX Cross P Andrews H Jacobs DM Small S Bell K Merchant C Lantigua R Costa R Stern Y Incidence of AD in African-Americans, Caribbean Hispanics, and Caucasians in northern Manhattan Neurology 2001 56 49 56 10.1212/WNL.56.1.49 11148235

[B34] Lobo A Saz P Marcos G Día J-L De-la-Cámara C Ventura T Montañés JA Lobo-Escolar A Aznar S The ZARADEMP Project on the incidence, prevalence and risk factors of dementia (and depression) in the elderly community: II Methods and first results Eur J Psychiatry 2005 19 40 54

[B35] Pluijm SM Bardage C Nikula S Blumstein T Jylha M Minicuci N Zunzunegui MV Pedersen NL Deeg DJ A harmonized measure of activities of daily living was a reliable and valid instrument for comparing disability in older people across countries J Clin Epidemiol 2005 58 1015 1023 10.1016/j.jclinepi.2005.01.017 16168347

[B36] Lobo A Lopez-Anton R De-la-Camara C Quintanilla MA Campayo A Saz P Non-cognitive psychopathological symptoms associated with incident mild cognitive impairment and dementia, Alzheimer's type Neurotox Res 2008 14 263 272 10.1007/BF03033815 19073431

[B37] Ganguli M Dodge HH Shen C DeKosky ST Mild cognitive impairment, amnestic type: an epidemiologic study Neurology 2004 63 115 121 10.1212/01.WNL.0000132523.27540.81 15249620

[B38] Petersen RC Mild cognitive impairment as a diagnostic entity J Intern Med 2004 256 183 194 10.1111/j.1365-2796.2004.01388.x 15324362

[B39] Winblad B Palmer K Kivipelto M Jelic V Fratiglioni L Wahlund LO Nordberg A Backman L Albert M Almkvist O Mild cognitive impairment–beyond controversies, towards a consensus: report of the International Working Group on Mild Cognitive Impairment J Intern Med 2004 256 240 246 10.1111/j.1365-2796.2004.01380.x 15324367

[B40] Carrillo MC Bain LJ Frisoni GB Weiner MW Worldwide Alzheimer's disease neuroimaging initiative Alzheimers Dement 2012 8 337 342 10.1016/j.jalz.2012.04.007 22748939

