# Supplementary material for: COSMIC (Cohort Studies of Memory in an International Consortium): An international consortium to identify risk and protective factors and biomarkers of cognitive ageing and dementia in diverse ethnic and sociocultural groups
Source: BMC Neurol. 2013 Nov 6;13:165. doi: 10.1186/1471-2377-13-165 (PMC3827845; doi:10.1186/1471-2377-13-165)
Supplement: Additional file 1 — Protocol for harmonising education across COSMIC member studies participating in the first project. [file 1471-2377-13-165-S1.docx]

**Additional file 1 Protocol for harmonising education across COSMIC member studies participating in the first project**

**COSMIC education categories**

1. Less than high school completion
2. High school completion
3. Technical or college diploma

4. University degree

**Table. Recoding of original education data into COSMIC categories**

| **Study** | **Original values used** | **Original value** → **COSMIC category** |
| --- | --- | --- |
| CLAS | Years | < 12 → 1  12 → 2  13-15 → 3  > 15 → 4 |
| EAS | 1. None 2. High school diploma/GED 3. Bachelors 4. Masters 5. Doctorate 6. Other | 1 → 1  2 → 2  3 → 4  4 → 4  5 → 4  6 → 3 |
| ESPRIT | 1. < 5th grade 2. 5th grade 3. 6th to 9th grade 4. Technical 9th grade 5. College 6. College graduate (including technical)   7. University | 1 → 1  2 → 1  3 → 2  4 → 2  5 → 2  6 → 3  7 → 4 |
| HK-MAPS | Years | < 12 → 1  12 → 2  13-14 → 3  > 14 → 4 |
| MAS | 1. Primary school 2. Incomplete high school 3. Completed high school 4. Incomplete tertiary 5. Completed tertiary 6. Incomplete high school + certificate/diploma 7. Completed high school + certificate/diploma | 1 → 1  2 → 1  3 → 2  4 → 2  5 → 4  6 → 3  7 → 3 |
| MoVIES | 1. Graduate/professional 2. College graduate 3. Partial college 4. Trade/technical 5. High school graduate 6. Partial high school 7. 6^th^-9^th^ grade 8. < 6^th^ grade | 1 → 4  2 → 3  3 → 2  4 → 3  5 → 2  6 → 1  7 → 1  8 → 1 |
| PATH | 1. Primary and secondary: 2. Some primary 3. All of primary 4. Some secondary 5. Three/four years of secondary (intermediate certificate) 6. Five/six years of secondary (higher school certificate) 7. Post-secondary schooling: 8. Trade certificate/apprenticeship 9. Technicians certificate/advanced certificate 10. Certificate other than above 11. Associate diploma 12. Undergraduate diploma 13. Bachelors degree 14. Postgraduate diploma/certificate 15. Higher degree | 1 → 1  2 → 1  3 → 1  4 → 1  5 → 2  1 → 1 if A = 1-4  1 → 2 if A = 5  2 → 3  3 → 3  4 → 3  5 → 3  6 → 4  7 → 4  8 → 4 |
| SLASI/II | 1. None 2. Primary 3. Secondary or ITE 4. Pre-university or polytechnic 5. University   Years | 1 → 1  2 → 1  3 → 1 if Years < 12 or missing  3 → 2 if Years = 12  3 → 3 if Years > 12  4 → 3  5 → 4 |
| WHICAP | Years | < 12 → 1  12 → 2  13-15 → 3  > 15 → 4 |
| ZARADEMP | 1. None 2. Less than primary 3. Primary 4. Less than technical formation 5. Technical formation 6. Less than high school 7. High school 8. College diploma 9. University degree | 1 → 1  2 → 1  3 → 1  4 → 1  5 → 3  6 → 1  7 → 2  8 → 3  9 → 4 |
